# Hospital Annual Meetings

**Published:** 1919-05-24

**Authors:** 


### HOSPITAL ANNUAL MEETINGS.

### Royal Waterloo Hospital.

The Lord Mayor (Sir Horace Marshall) presided over the annual Court of Governors, held at the Mansion House. In-patients, it was reported, numbered 833, compared with 870 in 1917, while out-patients had risen from 5,326 to 6,484, with 21,068 attendances. Income from all sources during 1918 was £12,116, and expenditure, including a payment of £454 towards the maintenance of the convalescent home in the Isle of Wight, totalled £10,947.

### Bolingbroke Hospital, Wandsworth.

The Governors, at their annual meeting, reported that during the past year 735 civilian and seventy-three military patients had been cared for in the wards. The number of out-patients was 4,562, and their attendances 28,276. The financial position of the hospital caused anxiety, and it was feared that unless the position materially improved it would be necessary to curtail the work the hospital is doing. The overdraft at the bank at the end of last year amounted to £7,157. The institution had no convalescent home of its own, and an urgent appeal was made for presents of letters for convalescent homes. A large number could be used annually for highly deserving cases.

### Hampstead General Hospital.

The Grand Duke Michael of Russia, who presided over the annual Court of Governors, stated that during the war over 1,000 soldiers had been cared for at the institution, the number treated in 1918 being 296. Last year had been the busiest one in the history of the hospital, the daily average of beds occupied reaching 118. Nevertheless, it was a period of great anxiety, and in spite of careful supervision and economy, the cost per in-patient showed an increase of 11 per cent. on the previous year. Over 1,450 in-patients were cared for, as compared with 1,206 in 1917. Gross income amounted to £18,108, while expenditure totalled £20,277. The total debt of the hospital at the end of last year was £5,134.

### Queen's Hospital for Children.

In the absence of Lord William Cecil, Mr. Joseph Mellor presided over the annual Court of Governors. The institution, he said, had been of national service inasmuch as it had saved the lives of a large number of children. An appeal was being made for a sum of £50,000, with which to erect a new out-patient department and an isolation block. They would complete the scheme of enlargement commenced in 1902, and were vital to the proper and efficient working of the hospital. The present out-patient department was opened in 1874, and in regard to the isolation wards a makeshift arrangement had had to serve for many years. In-patients, during the past year, numbered 1,481, and out-patients 53,119, with over 107,000 attendances. Income amounted to £20,227, while expenditure rose to £21,709, showing a balance on the wrong side of £1,482.

### Grosvenor Hospital for Women.

Mr. Chas. G. Arbuthnot presided over the annual Court of Governors of the Grosvenor Hospital for Women, Vincent Square, S.W., which treated 335 in-patients and 1,243 out-patients, with 6,664 attendances, during 1918. There was a daily average of twenty patients at the institution, and 305 operations were performed. Despite the exercise of strict economy, the cost per bed had gone up, and new annual subscribers upon whom the Committee depended so much to carry on the work were urgently needed. The anxiety of the Committee would have been greater had it not been for the receipt of legacies to the amount of nearly £350. As it was, the excess of expenditure over income amounted to £283.

### Victoria Hospital for Children.

At a Court of Governors it was reported that 209 in-patients and 2,800 new out-patients, whose attendances totalled 12,746, had been cared for at the institution during the quarter ended March 31 last. The income for the three months was £4,466, while expenditure amounted to £2,718. One of the wards at the hospital had been closed on account of the shortage of nurses.

### St. John's Hospital.

The Earl of Chesterfield, the President, who occupied the chair at the annual Court of Governors, said that for the last three or four years, in fact, since the war started, the expenditure had been increasing by leaps and bounds out of all proportion to the income. Two wards had been closed for some time owing to want of funds. That in itself reduced the number of poor people suffering from skin diseases who might have been cared for and cured. Extra accommodation both for patients and staff was urgently needed. So, too, was the large amount of money required to supply and maintain it. Annual subscriptions had never yet reached £1,000 a year.

### South London Hospital for Women.

The Marchioness of Londonderry, presiding over the annual meeting held at Londonderry House, said the private wards continued to prove a great boon, and would, she hoped, be extended, since they supplied a great want and were better than nursing homes. In-patients last year numbered 1,173, of whom 273 were cared for in the private wards. The number of out-patients was 7,386, as compared with 7,095 in 1917, attendances totalling 31,416, against 28,248 a year previously. Great difficulty was experienced last year owing to the shortage of nurses. The present accommodation was inadequate to meet the increasing demand on the hospital, and the need for the extension of the building became more and more apparent.

### Alexandra Hospital.

At the annual general meeting it was reported that during 1918 122 children were cared for, of whom 35 had been discharged cured and 5 discharged improved. The average number of beds in daily occupation was 53 in the hospital and 22 at the country branch at Clandon, in Surrey. New out-patients numbered 44. Annual subscriptions were slightly less than in 1917. Donations, however, rose from £450 to £527. The hospital, which now received patients sent by the London County Council under the National Health Act, possessed 90 beds—68 in London and 22 in the country.

